# Tuberculosis Beyond the Lungs: A Pictorial Review of Key Diagnostic Imaging Insights

**DOI:** 10.7759/cureus.81256

**Published:** 2025-03-26

**Authors:** Mateo Zapata Naranjo, Juan D Ayala Torres, Alma Tatiana Suarez Poveda, Vanessa García, Milena Alcazar Paternina

**Affiliations:** 1 Radiology, Universidad de Antioquia, Medellín, COL

**Keywords:** disseminated tuberculosis, extrapulmonar tuberculosis, general radiology, tuberculosis, unusual cases

## Abstract

Tuberculosis (TB) remains a significant global health challenge, traditionally associated with pulmonary manifestations. However, extrapulmonary tuberculosis (EPTB) accounts for a substantial portion of TB cases, particularly in immunocompromised patients. EPTB can affect virtually any organ system and often mimics other infectious, inflammatory, or neoplastic conditions, making diagnosis particularly challenging. This pictorial review aims to illustrate the broad spectrum of imaging findings in EPTB using selected, confirmed cases involving hepatic, splenic, adrenal, pancreatic, genitourinary, lymphatic, gastrointestinal, cardiovascular, musculoskeletal, and central nervous system sites.

Magnetic resonance imaging (MRI) and computed tomography (CT) are highlighted for their diagnostic capabilities, with MRI offering superior soft tissue contrast and CT providing high-resolution evaluation of organ involvement and guiding tissue sampling. Each case presented is supported by microbiological, histopathological, or molecular confirmation, reinforcing the importance of correlating radiologic features with definitive diagnostic tools. By enhancing familiarity with the diverse radiologic appearances of EPTB, this review seeks to improve diagnostic confidence and facilitate timely clinical decision-making in complex cases.

## Introduction and background

Tuberculosis (TB) is an infectious disease classically associated with pulmonary involvement but may manifest atypically in various organs, complicating diagnosis. These unusual presentations are often related to immunosuppressive states that impair cell-mediated immunity, facilitating both primary infection and reactivation of latent infections. Significant predisposing factors include HIV, immune-mediated inflammatory diseases treated with tumor necrosis factor (TNF)-alpha inhibitors, prolonged steroid use, organ transplantation, malnutrition, diabetes, smoking, and excessive alcohol consumption [1-4]. Clinical manifestations and imaging findings include extrapulmonary tuberculosis (EPTB) (pleural, osseous, articular, abdominal), miliary pulmonary forms, central nervous system (CNS) involvement, and complications such as abscesses in solid organs and cutaneous manifestations [5-9]. Early identification of these atypical forms through a diagnostic approach combining imaging, laboratory tests, and clinical evaluation is critical to ensuring effective treatment and improving clinical outcomes.

EPTB pathogenesis involves hematogenous or lymphatic spread of *Mycobacterium tuberculosis* from a primary focus, commonly the lungs [6]. Host immunosuppression, whether due to HIV, diabetes, or immunomodulatory therapies, plays a crucial role in reactivation and dissemination [2]. Prognosis varies significantly based on organ involvement; for instance, CNS and pericardial TB are associated with high morbidity and mortality, whereas abdominal forms may have favorable outcomes with timely treatment [2,6].

Computed tomography (CT) and magnetic resonance imaging (MRI) are pivotal in the evaluation of EPTB due to their ability to delineate anatomical structures, detect early tissue changes, and identify complications. MRI offers superior soft tissue contrast and is particularly useful in CNS, musculoskeletal, and abdominal TB. CT provides rapid evaluation of thoracic, abdominal, and osseous involvement, often guiding biopsy and management [6-8].

A literature review was conducted using PubMed, Scopus, and Google Scholar. Search terms included "extrapulmonary tuberculosis," "tuberculous abscess," "MRI TB," "CT TB imaging," and "atypical TB presentation." Filters were applied to select articles published in English from 2000 to 2024. Case reports, reviews, and pictorial essays with confirmed diagnoses were prioritized.

## Review

Methods

We conducted a narrative pictorial review based on a literature search and institutional imaging archives. Articles were identified through PubMed, Scopus, and Google Scholar using terms such as "extrapulmonary TB," "MRI TB," and "tuberculous abscess." Filters included English language and publication dates between 2000 and 2024. Selected cases included patients with imaging-confirmed EPTB and histopathological or microbiological confirmation. Informed consent was obtained for all patient images. This review emphasizes radiological features and patterns, aiming to support diagnostic recognition in clinical practice.

EPTB by organ system

Hepatic TB

**Figure 1:**
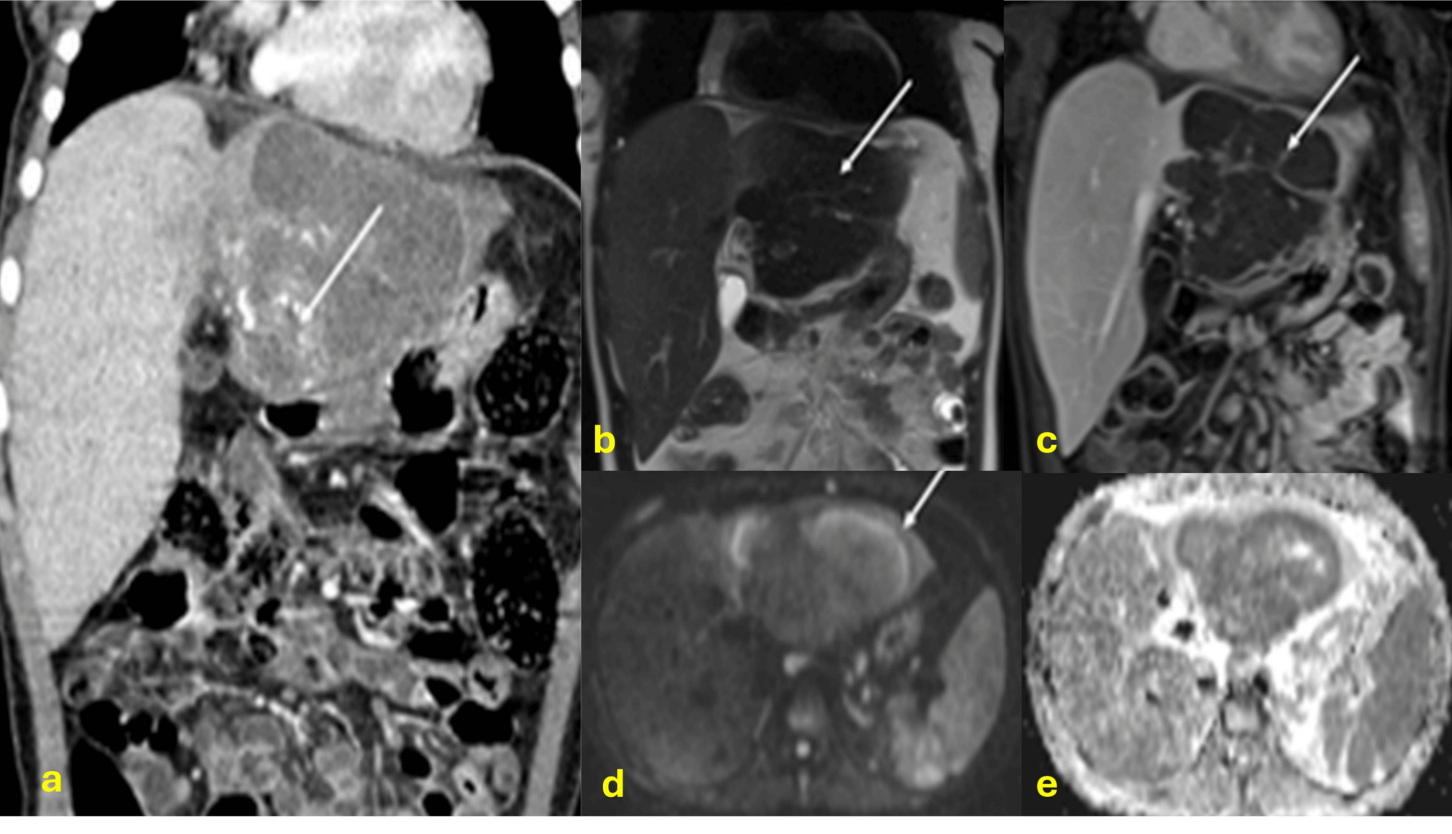
Hepatic tuberculoma CT: computed tomography; MRI: magnetic resonance imaging (a) Coronal contrast-enhanced abdominal CT scan in the venous phase showing a hypovascular mass with calcified areas (arrow) replacing the left hepatic lobe. (b) MRI showing a markedly T2-hypointense mass in the left hepatic lobe (arrow in b) with septal enhancement (arrow in c) and peripheral diffusion restriction (arrow in d and e) due to parenchymal compression. Diagnosis confirmed by surgical pathology after left hepatectomy showing caseating granulomas and positive Ziehl-Neelsen staining

Hepatic TB is a rare manifestation, accounting for less than 1% of TB infections. Although it can occur at any age, it is more frequently observed in young adults. The macronodular form may present as multiple lesions ranging from 1 to 3 cm or, in some cases, as a large tumorlike mass. These macronodular lesions have been referred to as tuberculomas, pseudotumoral TB, or tuberculous abscesses. Imaging characteristics are nonspecific and can mimic pyogenic abscesses, metastases, or primary tumors like hepatocellular carcinoma, fibrosarcoma, or cholangiocarcinoma. On CT scans, these lesions may enhance depending on their stage, while on MRI, their activity can influence contrast uptake and signal characteristics in T2 and diffusion-weighted sequences [10-13]. Fibrosis-dominant cases exhibit low T2 signal intensity and restricted enhancement confined to fibrotic septa (Figure 1).

Splenic TB

**Figure 2:**
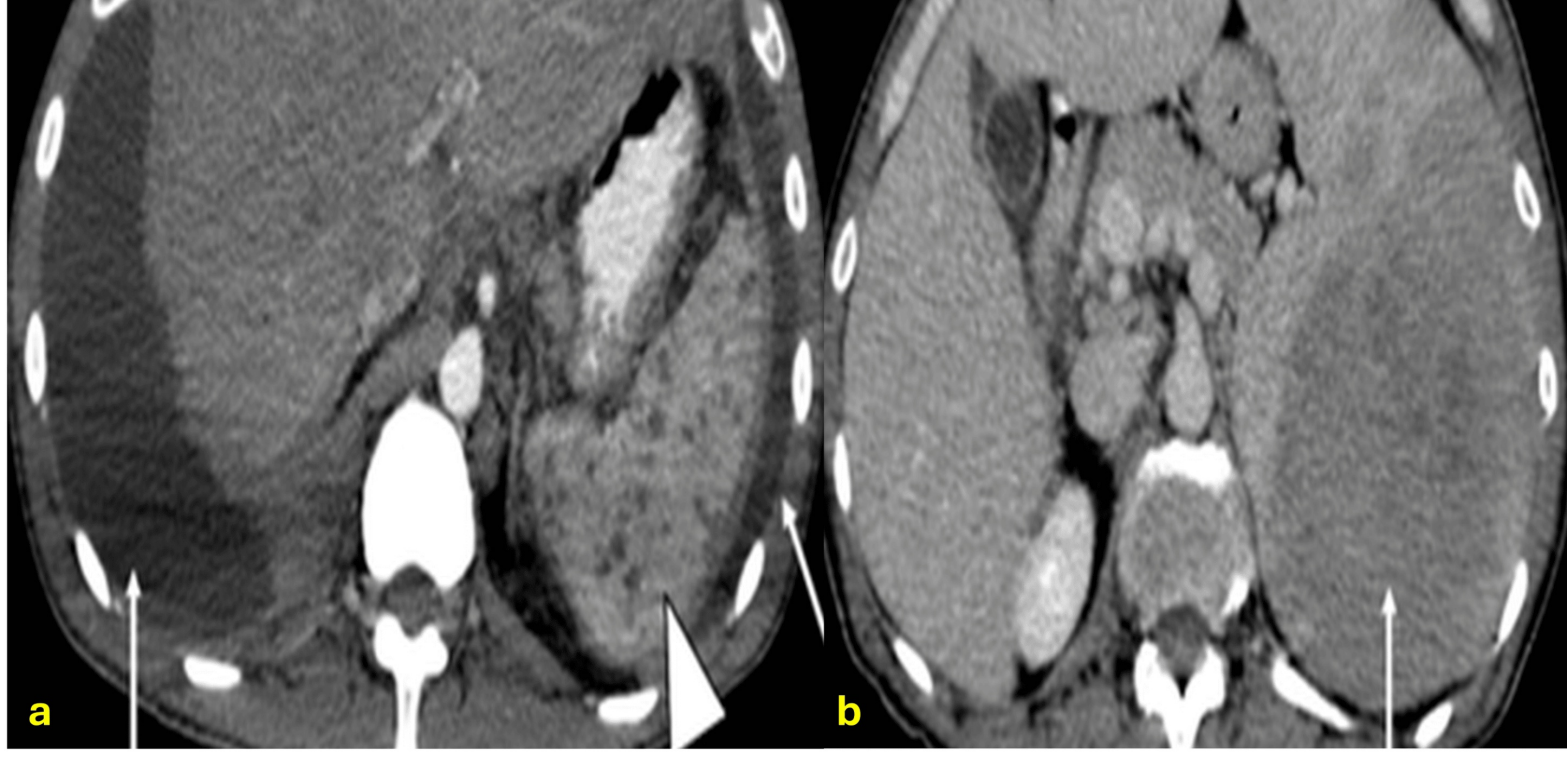
Splenic tuberculosis in two different patients CT: computed tomography; PCR: polymerase chain reaction (a) Axial CT scan of a patient with disseminated tuberculosis showing multiple, well-defined, hypodense, circular lesions distributed throughout the splenic parenchyma in a micronodular pattern (arrowhead). Note the bilateral pleural effusion with associated pleural thickening (arrows). (b) Axial contrast-enhanced CT scan of another patient showing a large, heterogeneous mass occupying most of the splenic parenchyma, consistent with macronodular tuberculosis involvement (arrow). Diagnosis confirmed by ultrasound-guided fine-needle aspiration (FNA) with positive PCR for *Mycobacterium tuberculosis*

Splenic involvement is typically seen in disseminated TB, with diagnostic imaging sensitivity around 45% [14,15]. It commonly presents as multiple microabscesses but may also appear as larger tuberculomas (macronodular form) [15,16]. CT and MRI findings typically show hypodense lesions with ring enhancement on CT and nodular late enhancement in some cases. Differential diagnoses include Kaposi sarcoma, metastases, lymphoma, and pyogenic abscesses (Figure 2) [16,17].

Adrenal Abscess

**Figure 3:**
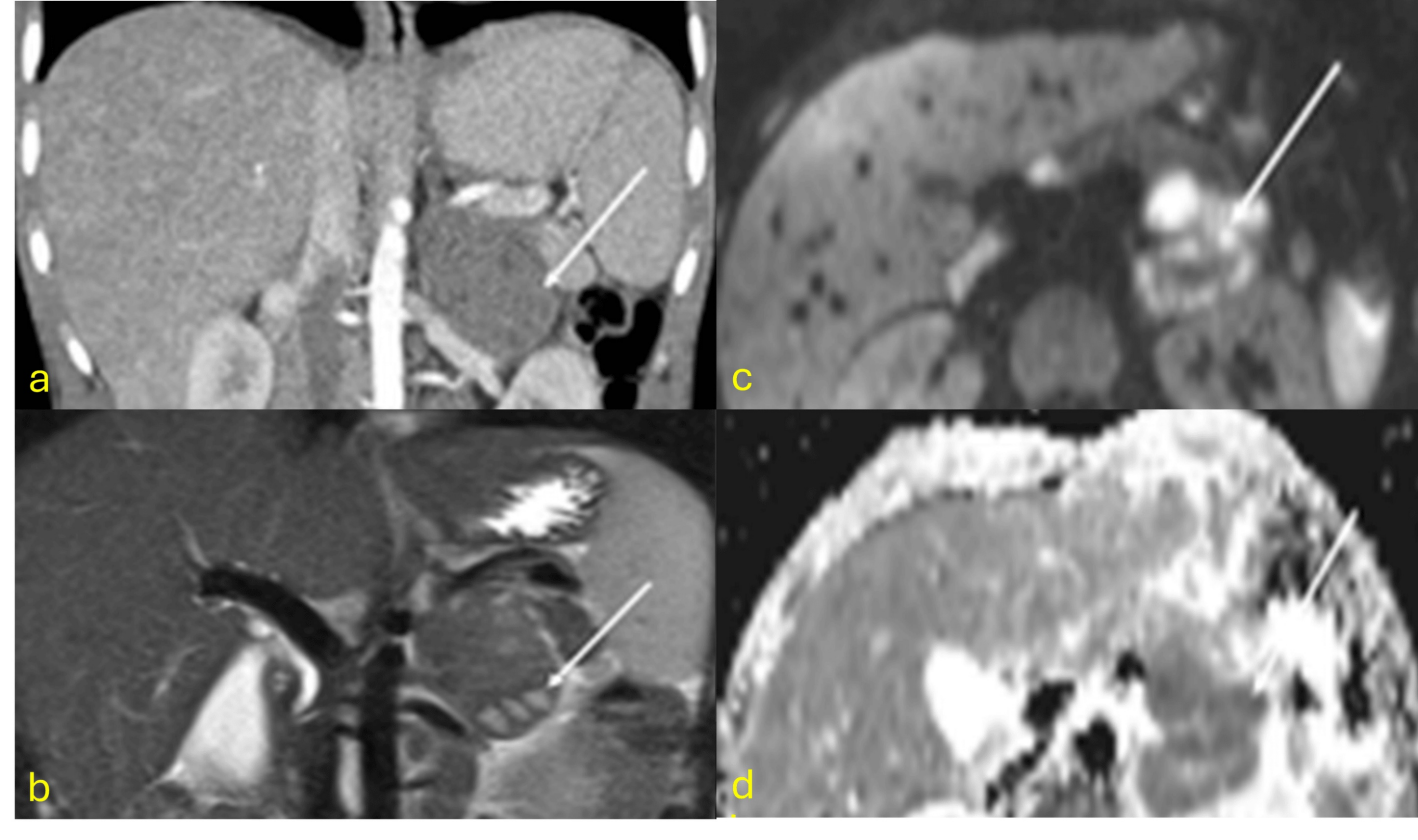
Tuberculous abscess in the left adrenal gland CT: computed tomography; MRI: magnetic resonance imaging (a) Coronal contrast-enhanced CT scan showing a 5 cm hypodense mass in the left adrenal gland with no post-contrast enhancement, in a patient with active pulmonary tuberculosis. (b) T2-weighted MRI and (c and d) diffusion-weighted MRI showing areas of high signal intensity on T2-weighted images and marked diffusion restriction, indicative of crypts (arrows). Diagnosis confirmed by CT-guided biopsy revealing necrotizing granulomatous inflammation and positive GeneXpert MTB/RIF assay

Adrenal TB occurs in approximately 6% of active TB patients [18]. During the active phase, adrenal enlargement (unilateral or bilateral) with preserved contours is common, often associated with necrotic or calcified central areas. Chronic stages result in adrenal atrophy and dystrophic calcifications. Adrenal TB is the leading cause of Addison’s disease in endemic regions [19]. On MRI, granulomas and caseous necrosis are evident as areas of high T2 signal intensity with restricted diffusion and ring enhancement, although calcifications are better identified via CT. Caseous necrosis is indicative of active TB, while fibrosis and calcifications suggest chronic lesions (Figure 3) [20].

Pancreatic Tuberculoma

**Figure 4:**
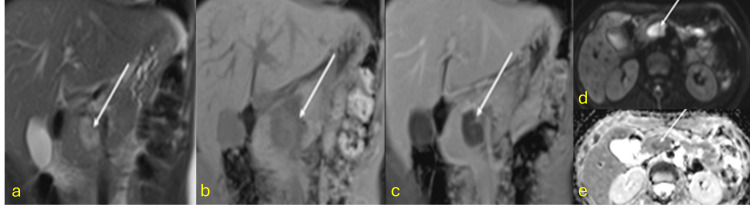
Pancreatic tuberculoma (a) Coronal T2 HASTE image showing a 3 cm mass in the pancreatic head with intermediate signal intensity (arrow), well-defined margins, and no ductal dilation. (b) Pre- and post-contrast coronal T1 fat-saturated images showing an intermediate signal mass (b) with no post-contrast enhancement (c) (arrow) and diffusion restriction (arrow in d and e). Diagnosis confirmed by endoscopic ultrasound-guided biopsy with histopathology showing caseating granulomas and positive culture for *Mycobacterium ​​​​tuberculosis*

Pancreatic TB is extremely rare [21]. Isolated pancreatic TB requires ruling out HIV infection or other predisposing conditions, as the pancreas is inherently resistant to mycobacterial colonization due to enzymatic activity [22]. The head and neck of the pancreas are the most commonly affected areas. Radiologically, pancreatic TB presents in three patterns: mass-forming (most common), diffuse, and micronodular. MRI findings include hypointense lesions on T1, hyperintense on T2, heterogeneity, peripheral enhancement, and sometimes septal enhancement, giving a multilocular appearance. Biopsy via endoscopic ultrasound is considered the most effective diagnostic method (Figure 4) [23,24].

Multifocal Infundibular Stenosis

**Figure 5:**
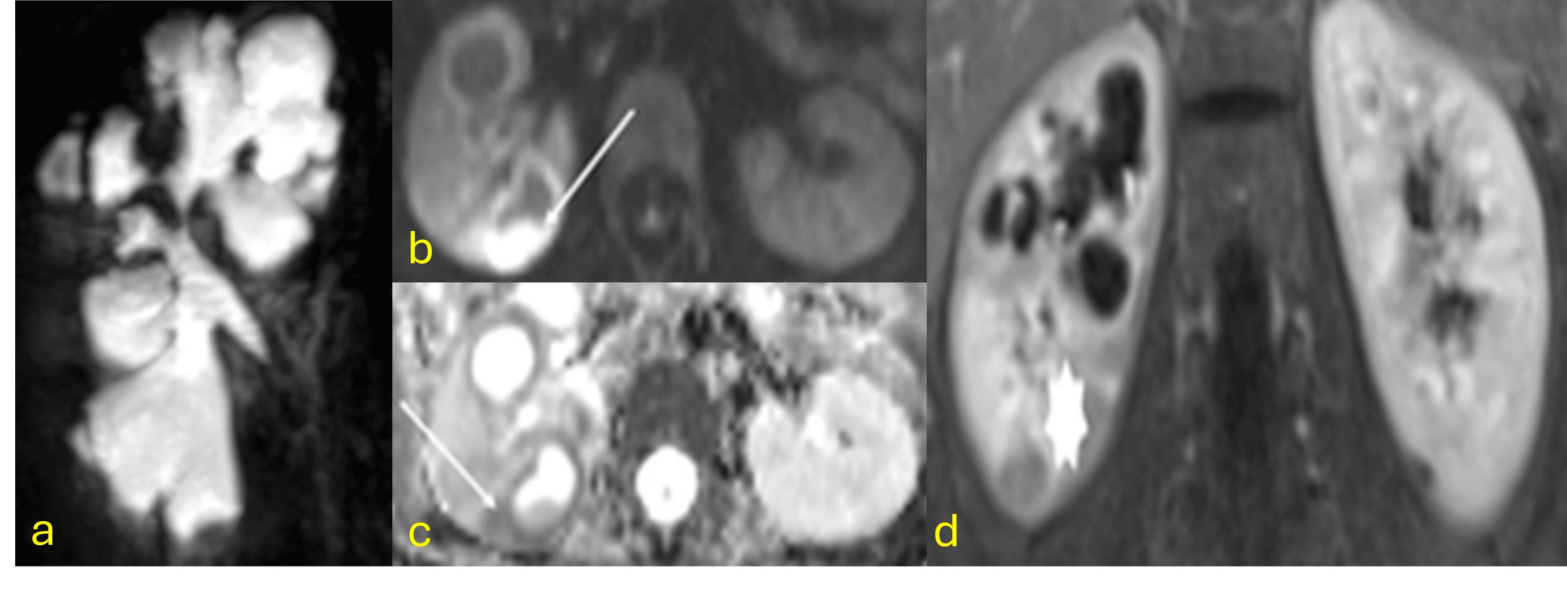
Multifocal infundibular stenosis (a) A patient under evaluation for right hydronephrosis. T2-weighted imaging shows multiple asymmetric infundibular strictures with caliectasis. The ureter is not involved. (b and c) Diffusion restriction is observed in the calyceal walls and dependent contents (arrows). (d) Post-contrast coronal image showing areas of focal pyelonephritis (asterisk). Diagnosis confirmed by urine lipoarabinomannan (LAM) antigen test and positive urine culture for *Mycobacterium ​​​​​tuberculosis*

Focal or diffuse caliectasis arises from stenosis of the pelvicalyceal or infundibular regions. Uneven caliectasis with urothelial thickening is a diagnostic hallmark of renal TB. Multifocal infundibular stenosis produces focal hydronephrosis. Major calyx involvement can lead to dilatation of minor calyces, often containing necrotic material or calcifications. MRI is superior for characterizing tissue pathology (Figure 5) [25,26].

Lymphatic TB

**Figure 6:**
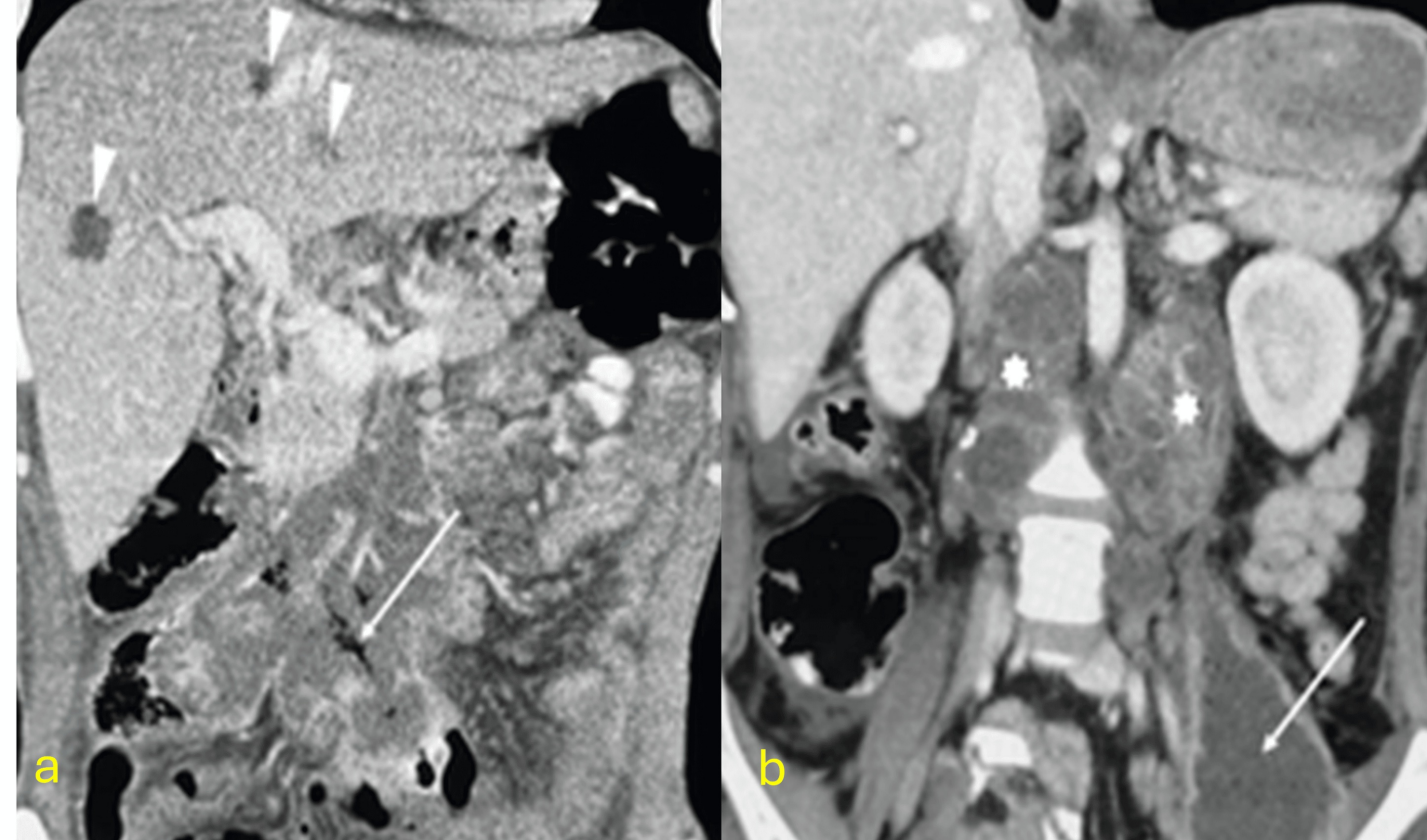
Abscessed lymph node tuberculosis CT: computed tomography (a) Coronal contrast-enhanced CT reconstructions showing mesenteric adenopathy conglomerates with hypodense areas and rim enhancement. Air bubbles indicate a fistula to the colon (arrow). Hepatic abscesses are also noted (arrowhead). (b) Retroperitoneal adenopathy (asterisks) compromising the left psoas muscle with abscess formation. Diagnosis confirmed by CT-guided biopsy of mesenteric lymph nodes showing granulomatous inflammation with caseation and positive GeneXpert MTB/RIF

The cervical lymph nodes are most frequently affected due to their proximity to the pulmonary parenchyma. Between 6% and 18% of peripheral tuberculous lymphadenitis cases also involve abdominal lymph nodes [27]. Commonly affected abdominal lymphatic groups include omental, mesenteric, and peripancreatic nodes. Clinical symptoms vary by site, potentially including abdominal pain, jaundice, portal thrombosis, and duodenal obstruction. Imaging reveals characteristic stages: initial lymphoid proliferation, caseous necrosis, and capsular degeneration leading to conglomerate abscess formation (Figure 6) [28-30].

Anorectal TB

**Figure 7:**
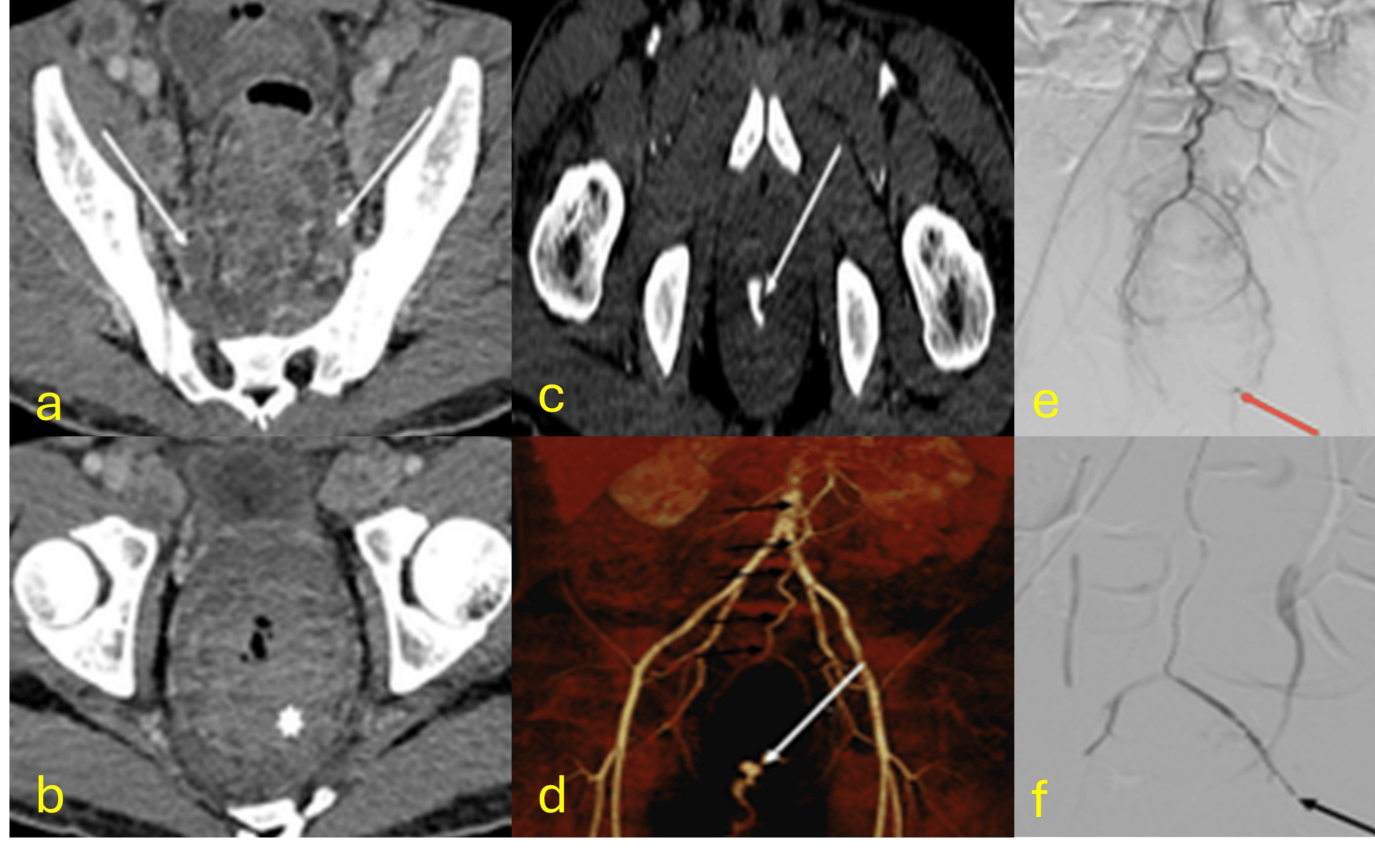
Anorectal tuberculosis with active arterial bleeding CT: computed tomography (a) Axial contrast-enhanced CT images showing hypodense pelvic adenopathies with rim enhancement (arrows). (b) The rectum exhibits marked wall thickening with a layered appearance (asterisk). Spontaneous drainage of a perianal abscess was positive for TB. (c and d) Later, the patient developed severe rectal bleeding. CT angiography shows active contrast extravasation (arrow in c) into the rectal lumen, with 3D reconstruction demonstrating the bleeding origin in the inferior mesenteric artery (arrow in d). (e and f) Selective angiography of the inferior mesenteric artery reveals bleeding from distal branches of the superior rectal artery (red arrow in e), which was embolized with two coils, successfully controlling the bleeding (black arrow in f). Diagnosis confirmed by rectal mucosal biopsy during colonoscopy revealing necrotizing granulomas and positive Ziehl-Neelsen staining

Anorectal involvement is extremely rare, occurring in only 1% of abdominal TB cases [31]. Lower gastrointestinal bleeding as a manifestation of disseminated TB is also infrequent. Diagnosis typically requires a colonoscopy and biopsy. Imaging shows concentric rectal wall thickening with significant diagnostic differentials (Figure 7) [32-34].

Tuberculous Aortitis

**Figure 8:**
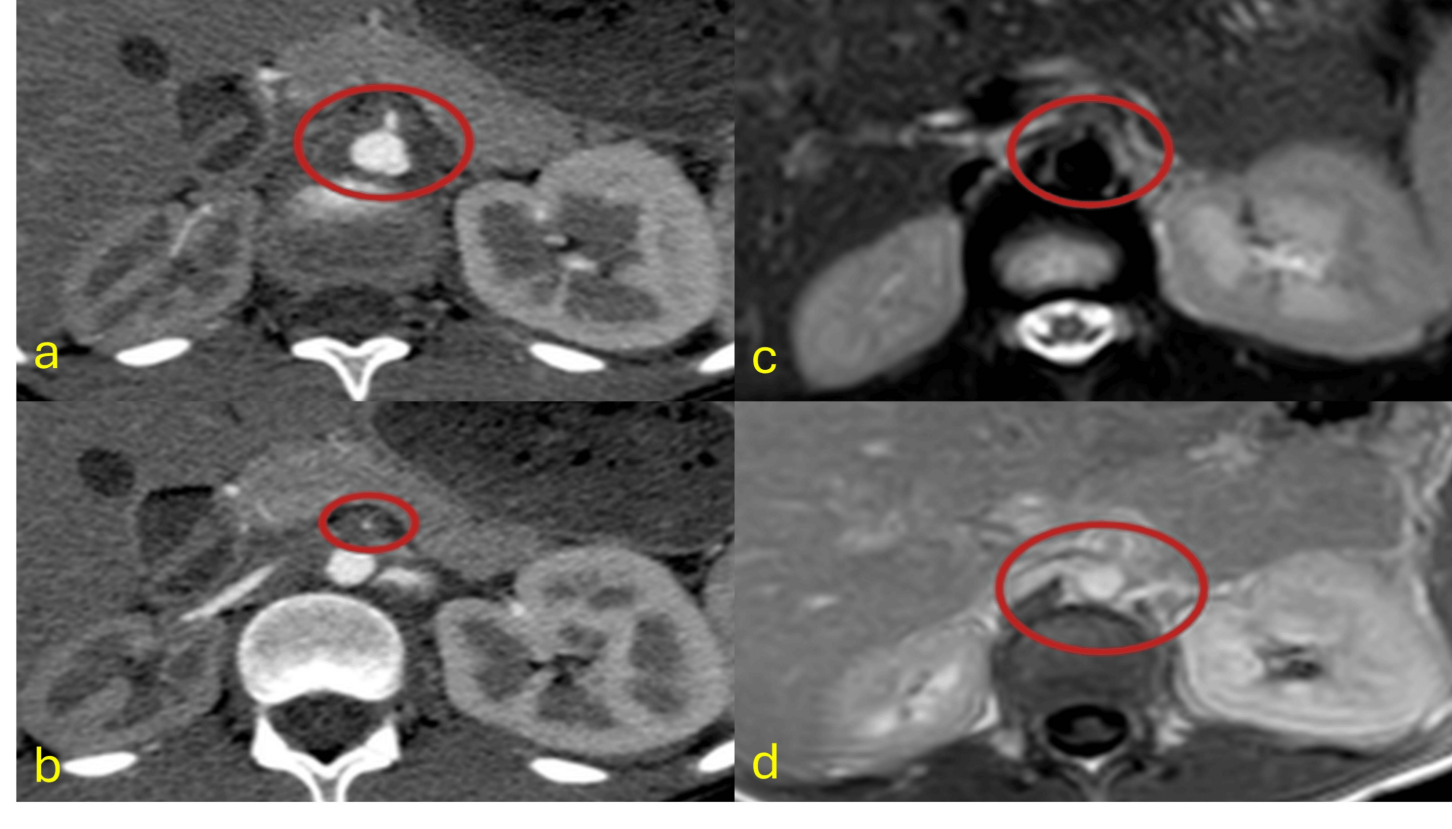
Tuberculous aortitis CT: computed tomography; MRI: magnetic resonance imaging; TB: tuberculosis; PCR: polymerase chain reaction (a and b) Axial contrast-enhanced CT angiography images showing a hypodense soft tissue component surrounding the suprarenal abdominal aorta and encroaching on the origin of the superior mesenteric artery, causing significant stenosis (red circles). Note the reduced size and altered enhancement pattern of the right kidney due to renal artery involvement. (c) Axial T2-weighted MRI with fat saturation shows predominantly hyperintense soft tissue with hypointense areas around the origin of the right renal artery (red circle). (d) Axial T1-weighted post-contrast MRI image demonstrates the same soft tissue involvement extending to the right renal artery (red circle). Diagnosis confirmed by CT-guided biopsy of periaortic soft tissue showing necrotizing granulomatous inflammation and positive TB-PCR

Tuberculous vasculitis is rare, presenting primarily as saccular aneurysms in disseminated TB cases. Secondary spread from infected lymph nodes is the predominant mechanism [35-37]. Imaging identifies aneurysms with adjacent soft tissue involvement. MRI demonstrates diffusion restriction in soft tissue components, aiding diagnosis (Figure 8) [36].

Tracheal TB

**Figure 9:**
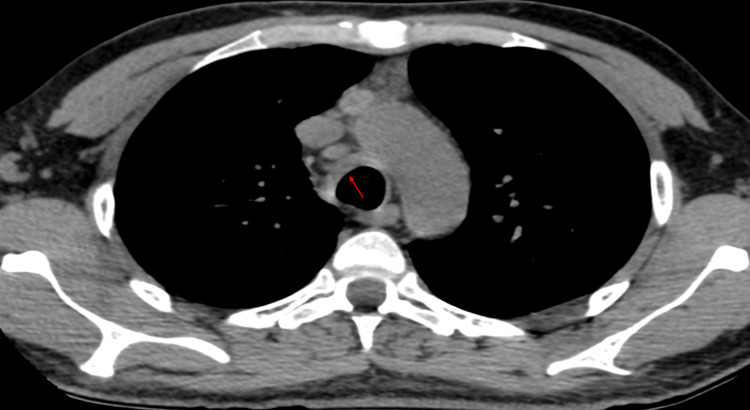
Tracheal tuberculosis CT: computed tomography Axial CT scan showing thickening of the right anterolateral tracheal wall in a patient diagnosed with tuberculosis (red arrow). Diagnosis confirmed by bronchoscopic biopsy of the tracheal wall demonstrating granulomatous inflammation and positive acid-fast bacilli

Airway structures are usually affected secondarily by infected lymph nodes or direct implantation. Rarely, isolated involvement occurs [38]. Manifestations include mediastinal adenopathy, tracheal stenosis, or fistula formation. CT is the preferred imaging modality (Figure 9) [38,39].

Pericardial TB

**Figure 10:**
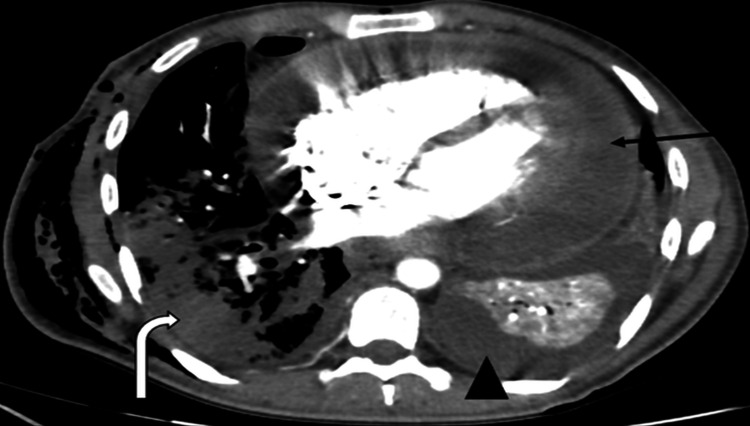
Pericardial tuberculosis ADA: adenosine deaminase; CT: computed tomography Axial contrast-enhanced thoracic CT angiography showing pericardial effusion with associated pericardial enhancement (black arrow), pleural effusion (arrowhead), and consolidations in the right lung parenchyma (curved arrow). Diagnosis confirmed by analysis of pericardial fluid, revealing high ADA levels and positive culture for *Mycobacterium tuberculosis*

Pericardial involvement, though rare, has high mortality. Common in HIV co-infected patients, it is the second most fatal TB manifestation after CNS involvement. Imaging findings include pericardial effusion, thickening, and enhancement (Figure 10) [40-42].

Musculoskeletal TB

**Figure 11:**
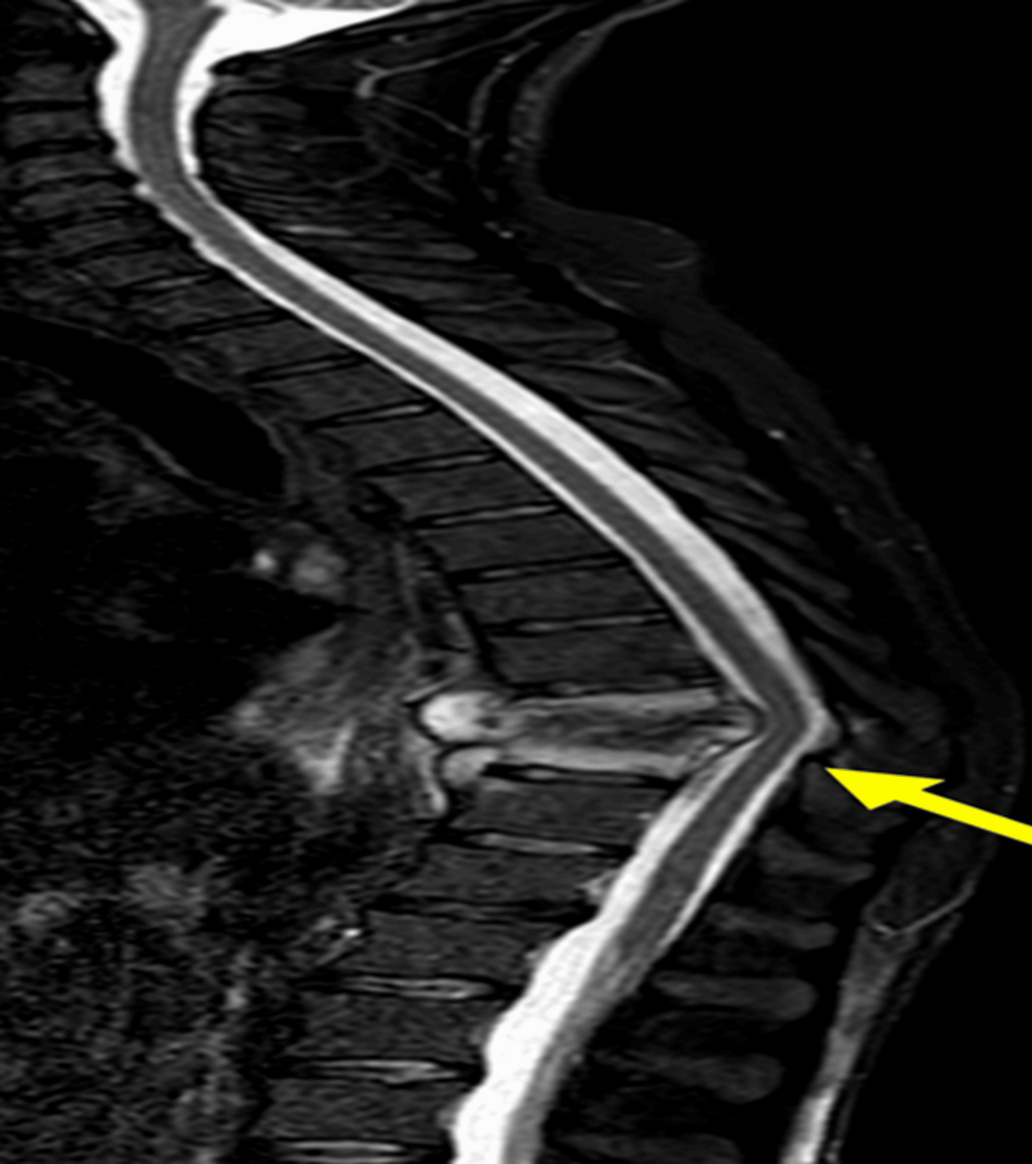
Spinal tuberculosis STIR: short tau inversion recovery; MRI: magnetic resonance imaging Sagittal STIR MRI sequence showing partial destruction of the T9 and T10 vertebral bodies, with edema and destruction of their endplates and the intervertebral disc due to T9-T10 spondylodiscitis (arrow) in a patient with disseminated tuberculosis and thoracolumbar pain. Diagnosis confirmed by MRI-guided vertebral biopsy showing caseating granulomas and culture-positive for *Mycobacterium tuberculosis*

**Figure 12:**
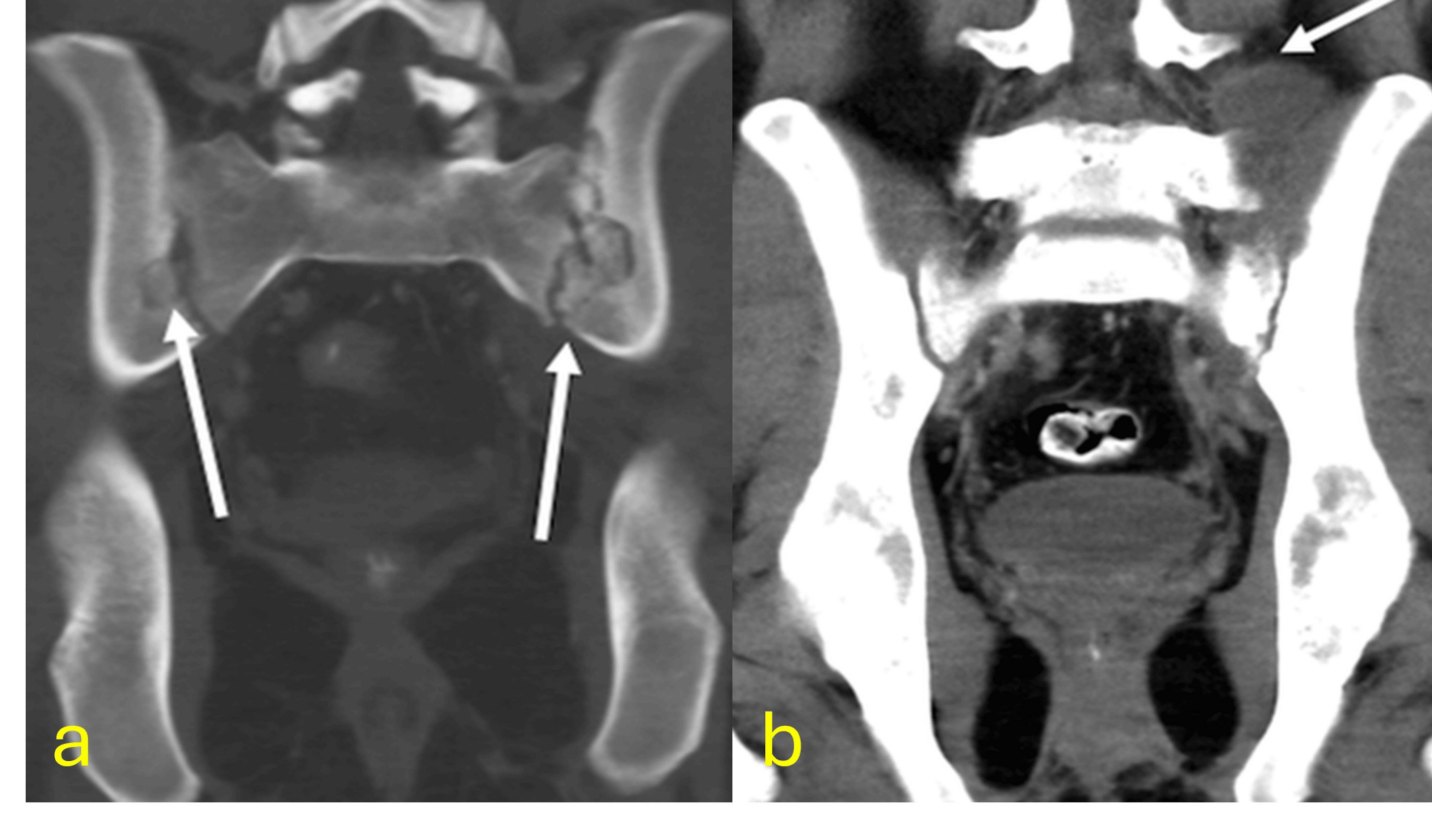
Tuberculosis of the sacroiliac joints with iliac muscle abscess CT: computed tomography (a) Coronal CT reconstruction in bone window showing erosions in both sacroiliac joints (arrows). (b) Coronal reconstruction in soft tissue window of the same patient shows an abscess in the left iliac muscle (arrow). Diagnosis confirmed by CT-guided biopsy of the iliac muscle abscess with histology showing necrotizing granulomatous inflammation

**Figure 13:**
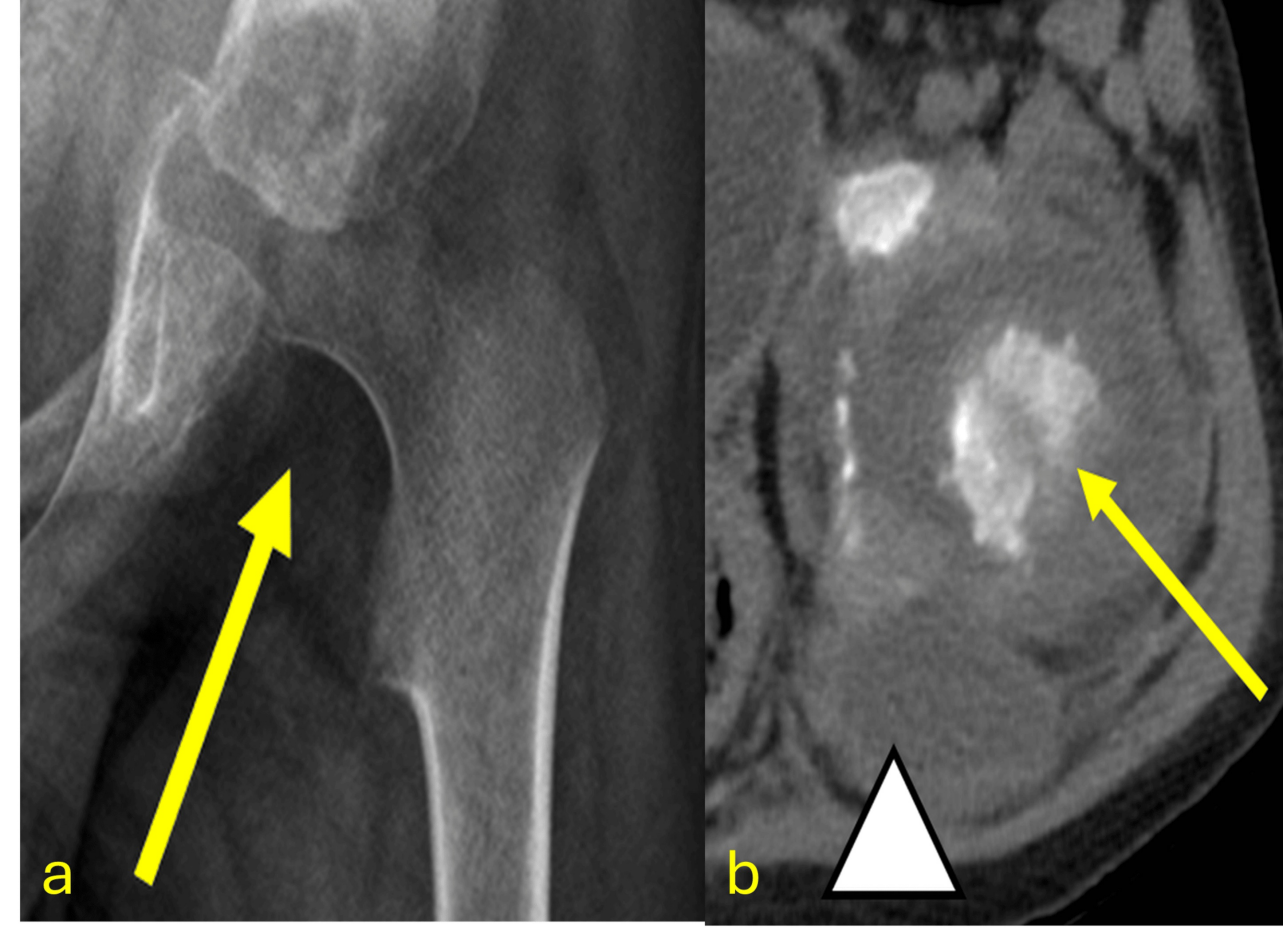
Pediatric patient with tuberculosis-related hip involvement CT: computed tomography; TB: tuberculosis; PCR: polymerase chain reaction (a) Anteroposterior pelvic X-ray shows increased radiolucency of the soft tissues in the left hip and loss of architectural integrity of the femoral head and neck surface (yellow arrow). (b) Axial noncontrast CT scan of the pelvis reveals destruction of the left femoral head (yellow arrow) with edema in adjacent soft tissues and an abscess in the anterior muscle plane of the left gluteus maximus (arrowhead). Diagnosis confirmed by surgical debridement and histopathology of the femoral head, revealing caseating granulomas and positive TB-PCR

Musculoskeletal TB affects 3%-10% of TB patients, primarily the spine (50%-70%). Clinical presentation is often delayed due to low suspicion. MRI reveals early inflammatory changes, abscesses, and spinal compression. Differential diagnoses include bacterial or fungal spondylodiscitis and metastases (Figures 11-13) [43-48].

CNS TB

**Figure 14:**
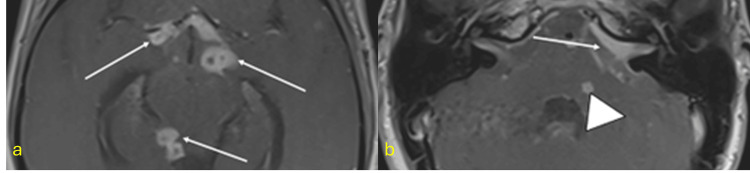
CNS tuberculosis ADA: adenosine deaminase; MRI: magnetic resonance imaging; CNS: central nervous system Axial T1-weighted post-contrast MRI images showing (a) nodular leptomeningeal enhancement in the basal cisterns (arrows) and (b) a tuberculoma in the left cerebellopontine angle (arrow) and a small tuberculoma within the cerebellar parenchyma (arrowhead). Diagnosis confirmed by cerebrospinal fluid (CSF) analysis showing lymphocytic pleocytosis, elevated ADA, and positive GeneXpert MTB/RIF

CNS TB, though rare (1%-3% of TB cases), has high mortality. MRI findings vary by lesion stage and include leptomeningitis, hydrocephalus, tuberculomas, abscesses, and cerebritis. Early diagnosis is crucial for improved outcomes (Figure 14) [49-55].

Discussion

This pictorial review illustrates the diverse manifestations of EPTB and reinforces the importance of cross-sectional imaging in diagnosis. The cases reflect the findings described in prior studies [6,10,14,22,43,49], confirming that imaging patterns such as necrotic lymphadenopathy, ring-enhancing lesions, and cold abscesses remain key diagnostic clues.

MRI and CT are the primary modalities for evaluating deep organ involvement, with MRI offering superior soft tissue characterization in CNS and musculoskeletal TB [20,43,49]. While ultrasound may be helpful for superficial structures and biopsy guidance, it lacks specificity for complex or deep lesions and was therefore not included in this review [6,14].

All cases in this series were confirmed by histopathology, microbiological culture, or molecular testing, underscoring the importance of correlating imaging with tissue diagnosis.

## Conclusions

EPTB poses a significant diagnostic challenge due to its atypical manifestations and resemblance to other diseases. Early identification relies on clinical suspicion supported by advanced imaging techniques such as MRI and CT. Recognizing specific radiological patterns and integrating them with clinical and laboratory findings helps differentiate TB from neoplastic or inflammatory conditions, while targeted biopsy remains essential for definitive diagnosis in complex cases. Given the high morbidity of forms like CNS TB and tuberculous pericarditis, timely detection is crucial for improving prognosis and guiding appropriate treatment.
